# Intracloacal Inoculation of Broiler Chickens with *Clostridium perfringens* Strains: Evaluation of Necrotic Enteritis Disease Development and Lymphoid Immune Responses

**DOI:** 10.3390/microorganisms11030771

**Published:** 2023-03-17

**Authors:** Carissa Gaghan, Kaitlin Gorrell, Khaled Taha-Abdelaziz, Shayan Sharif, Raveendra R. Kulkarni

**Affiliations:** 1 Department of Population Health and Pathobiology, College of Veterinary Medicine, North Carolina State University, Raleigh, NC 27607, USA; 2 Department of Animal and Veterinary Sciences, Clemson University, Clemson, SC 29634, USA; 3 Department of Pathobiology, Ontario Veterinary College, University of Guelph, Guelph, ON N1G 2W1, Canada

**Keywords:** necrotic enteritis, *Clostridium perfringens*, chickens, intracloacal infection, immune response, virulence

## Abstract

Necrotic enteritis (NE) is an economically important disease of chickens. We have recently shown that inflammatory responses in chickens inoculated orally with virulent *Clostridium perfringens* were spatially regulated. Here, we used previously virulence-characterized *netB*^+^ *C. perfringens* strains, avirulent CP5 and virulent CP18 and CP26, to assess the severity of NE and immune responses in broiler chickens when inoculated intracloacally. The results showed that CP18- and CP26-infected birds had a reduced weight gain and developed milder/less severe NE lesions, as determined by the gross lesions scores, suggesting a subclinical-grade infection. Gene expression analysis in infected birds revealed three statistically significant observations compared to uninfected-control: (1) Increased expression of anti-inflammatory/immunoregulatory interleukin (IL)-10/transforming growth factor (TGF)β in cecal tonsil (CT) and bursa of Fabricius in the CP18/CP26-infected groups. (2) Increased CT transcription of pro-inflammatory IL-1β, IL-6 and interferon (IFN)γ and decreased Harderian gland (HG) expression of IFNγ in the CP18/CP26-infected birds. (3) Increased HG or bursal expression of IL-4 and IL-13 in CP5-infected birds. Collectively, intracloacal *C. perfringens* inoculation seems to induce a highly regulated inflammatory response in the CT and other mucosal lymphoid organs and an intracloacal infection model may be useful in evaluating immune responses in chickens with subclinical NE.

## 1. Introduction

Necrotic enteritis (NE), caused by the NetB toxin-producing virulent strains of *Clostridium perfringens* type G, is a multifactorial disease of chickens [1]. In recent years, another toxin, TpeL, has also been shown to enhance the virulence in some *netB^+^ C. perfringens* [2,3,4]. The disease occurs in two forms: clinical and subclinical. While the clinical NE results in production losses associated with mortality and antibiotic treatment costs, the subclinical NE, which often goes undetected, results in a considerable reduction in bird performance, particularly in broiler populations. The global economic losses due to both clinical and subclinical NE, as of 2015, were estimated at around USD 6 billion/year [5]. Due to the growing concerns over the emergence of antimicrobial resistance, many countries have now placed a regulatory ban on the use of antibiotic feed supplements, which in turn has caused an increase in the incidences of NE in broiler flocks [6]. Ample research is now being conducted to study NE pathogenesis, immunity, and control, using several experimental NE reproduction models [7].

Several studies aimed at understanding the host immune responses against *C. perfringens* have used experimental NE reproduction models of commercial broiler chickens or genetically resistant/susceptible lines for *C. perfringens* infection [8,9]. For example, a previous study, using an experimental *C. perfringens* infection model of broiler chickens, showed an increased expression of immune response genes in the ileum and spleen of infected birds [10]. These included toll-like receptor (TLR) genes such as TLR1, TLR2, TLR4, TLR7, and TLR15, the TLR-pathway adaptor genes such as myeloid differentiation primary response 88 (MyD88), TIR-domain-containing adapter-inducing interferon-β (TRIF), as well as the chemokine interleukin (IL)-8. A subsequent study used an *Eimeria maxima* and *C. perfringens* co-infection model to infect two genetically disparate commercial broiler chicken lines (Ross and Cobb) and assessed the expression avian β-defensins (AvBD) genes in the crop, intestine, and spleen [11]. The findings showed that the Ross birds had a consistently higher expression of AvBD genes than the Cobb line, suggesting a role for these defensins in resistance against NE. Although the design and outcome of these experimental models have often depended on the objective of the research investigation, two important components that always have been considered are: (1) Including disease predisposing factors such as diet, *Eimeria* infection, or immunosuppression, and (2) *C. perfringens* inoculation via oral gavage route, for inducing either clinical [12] or subclinical [13] infections. Our previous work, using a dietary predisposition model, has shown that certain proteins secreted by virulent *C. perfringens* play an important role in immunity against NE, which included alpha toxin and tissue degrading, as well as metabolic, enzymes [14]. Subsequently, using *Eimeria* predisposition, several groups including us, have shown that oral inoculation of broiler chickens with virulent *C. perfringens* can induce elevated intestinal gene expression of TLR2, TLR15, and TLR21 receptors, and cytokines, such as interleukin (IL)-1β, IL-6, IL-8, IL-12, IL-10, interferon (IFN)γ, and transforming growth factor (TGF)β [15,16,17].

More recently, we used an experimental oral inoculation model of broiler chickens comprised of only dietary predisposition to test the virulence of different *C. perfringens* strains, as well as the strain-specific host immune responses [4]. The study used three *netB*^+^ *C. perfringens*, namely CP5 (*tpeL^-^*), CP18 (*tpeL^+^*), and CP26 (*tpeL^+^*), to find CP5 as avirulent, while CP18 and CP26 were virulent, producing ulcerative and necrotic lesions characteristic of clinical NE. Additionally, the evaluation of lymphoid tissue responses showed a marked pro-inflammatory response in the cecal tonsil (CT) and spleen and an immunoregulatory response in the bursa of Fabricius and harderian gland (HG) of birds infected with virulent strains. This observation indicated that immune responses against *C. perfringens* were spatially regulated and that the inflammatory response depended on the level of pathogen virulence. In another study, we also found that macrophages may be the cellular source of inflammatory cytokines induced in response to virulent *C. perfringens* [18]. In the present study, we asked if intracloacal inoculation of broiler chickens with the same *C. perfringens* strains (CP5, CP18, and CP26) and the dietary predisposition model used previously [4] would impact the severity of NE development and its associated mucosal and systemic lymphoid immune responses. To address this question, first, a previously established NE lesion scoring system [12], along with the body weight gain data, was used to assess the nature (clinical or subclinical) of NE. Next, the expression of TLR21, IL-1β, IL-6, IFNγ, IL-4, IL-10, IL-13, and TGFβ genes in lymphoid tissues (CT, bursa, HG, and spleen) was quantitated to evaluate both mucosal and systemic immune responses.

## 2. Materials and Methods

### 2.1. C. perfringens Strains

Three *netB^+^ C. perfringens* clinical isolates (strains CP5, CP18, and CP26) were generously provided by Dr. John F Prescott, University of Guelph, Canada. Isolates were grown and stored in cooked meat medium (CMM) (Oxoid, KS, USA). While the CP5 was found to be avirulent in our previous oral infection challenge experiments in broiler chickens [4,19], the CP18 and CP26 that carried an additional *tpeL* gene were found to be virulent [4].

### 2.2. Animals

All animal experiments and the procedures used in this research were approved by the Institutional Animal Care and Use Committee of North Carolina State University (IACUC protocol #19-077-A). A total of 75 commercial one-day-old broiler chicks that were not hatchery vaccinated against coccidiosis were generously provided by Perdue Farms Hatchery (Kenly, NC, USA). Chicks were reared for a period of 22 days in grouped floor pens on fresh litter at the Laboratory Animal Research (BSL-2) facility of the College of Veterinary Medicine, NC State University, and were given unlimited access to feed and water. Chickens were fed an antibiotic-free starter diet containing 20% crude protein for the first 10 days, followed by a wheat-based diet containing 10% fishmeal with a total crude protein content of 30% to dietarily predispose the birds to NE development. Individual body weights were taken on day 17 (pre-challenge) and day 22 (post-challenge, prior to necropsy) and % weight gain was calculated as (Day 22 weight − Day 17 weight)/Day 17 weight.

### 2.3. Intracloacal Inoculation

A pilot study was performed to evaluate the extent of inoculum reachability to different parts of the intestinal tract by administering a food dye intracloacally to 3-week-old broiler birds (*n* = 5) along with PBS control. The assorted food colors and the egg dye were purchased (McCormick, Hunt Valley, MD, USA) and the black dye was prepared by mixing 1-part blue food dye with 2.5 parts green dye and 3 parts red dye. With the bird held upside-down gently in one hand, a pre-lubricated 3-inch blunt-ended plastic canula attached to a pipette was passed gently through the cloaca approximately ½–1 inch to deliver 1mL of the dye solution [20]. The outside of the cloaca was gently tapped to stimulate movement of the vent (cloacal drinking) and the reverse peristalsis motion. Birds were then held in an upside-down position briefly before being put back into their pens. Birds were necropsied at about 1hr post-inoculation and the large intestine (colon-rectum and cecum) and small intestine (ileum, jejunum, and duodenum) were observed for the presence of black dye. This method of inoculation was later used in the experimental challenge model to inoculate birds with *C. perfringens* strains.

### 2.4. Experimental Challenge and Gross Pathology

Chickens were divided into five groups (*n* = 15) when they were 17 days of age. Three experimental groups were infected with *C. perfringens* strains, CP5, CP18, or CP26, and the infected chickens were kept in rooms separate from the uninfected (negative control) group of birds. For infection, *C. perfringens* strains were grown anaerobically in cooked meat medium (CMM) for 24 h at 37 °C, as described previously [4]. The fluid thioglycolate medium (FTG, Difco, Franklin Lakes, NJ, USA) was then inoculated with a 3% (*v/v*) inoculum from the *C. perfringens*-infected CMM and incubated at 37 °C for 15 h. Chickens were challenged intracloacally, when they were 18 days old, with 1mL of culture containing about 5.8–8 × 10^8^ CFU of *C. perfringens* twice daily for 4 consecutive days. The negative control birds received FTG only, the medium that was used for growing *C. perfringens*.

On the 22nd day of age, chickens were euthanized using Carbon dioxide and the gross lesions in the small intestine (duodenum to ileum) were scored as described previously [12]. Briefly, a score of 0 was given when no gross lesions were found; 1 for thin/friable walls or diffuse superficial but removable fibrin; 2 for focal necrosis or ulceration, or non-removable fibrin deposit with 1 to 5 foci of lesions; 3 for focal necrosis or ulceration, or non-removable fibrin deposit with 6 to 15 foci of lesions; 4 for focal necrosis or ulceration, or non-removable fibrin deposit with 16 or more foci of lesions; 5 for patches of necrosis 2 to 3 cm long; and a score of 6 was given when diffuse, extensive necrosis was observed. It is noteworthy here that no coccidia (*Eimeria*)-mediated intestinal predisposition was used in the present study’s experimental NE challenge model to avoid possible confounding effects of the Eimeria species on the expression profile of the immune genes.

### 2.5. Real-Time PCR

Tissues, namely, spleen, CT, bursa, and HG, were collected in RNAlater solution (Invitrogen, Carlsbad, CA, USA) from 8 chickens/group at necropsy and stored at −80 °C until processing. The birds showing NE lesions in the infected groups were preferentially included in the gene expression analysis to allow an effective evaluation of immune responses against the *C. perfringens* strains that produced NE of varying severities. Total RNA was extracted using a Bead Ruptor Elite Bead Mill Homogenizer (OMNI International, Kennesaw, GA, USA) using 1.4 mm Ceramic Beads (OMNI International, Kennesaw, GA, USA) suspended in TRIzol reagent (Invitrogen, Carlsbad, CA, USA) according to the manufacturer’s protocol, before being treated with a DNA-free Kit (Invitrogen, Carlsbad, CA, USA). Subsequently, cDNA synthesis was performed with 1000 ng of purified RNA using a High-Capacity RNA-to-cDNA kit (Applied Biosystems, Waltham, MA, USA) according to the manufacturer’s recommended protocol. The resulting cDNA was subsequently diluted 1:10 in nuclease-free water for real-time PCR analysis.

Quantitative real-time reverse-transcriptase PCR using SYBR Green was performed on diluted cDNA using a QuantStudio 6 Flex System and QuantStudio Real-Time PCR Software v1.7.2 (Applied Biosystems, Waltham, MA, USA). Briefly, each reaction involved a pre-incubation period of two min at 50 °C followed by two min at 95 °C, followed by 40–50 cycles of 95 °C for 10 sec, 55–64 °C for 5 sec, depending on the primers’ binding suitability, and the elongation step was 72 °C for 10 sec. Subsequent melt curve analysis was performed by heating to 95 °C for 15 sec, cooling to 60 °C for 1 min, and heating to 95 °C for 15 sec. Primers for the amplification of TLR21, IL-1β, IL-4, IFNγ, IL-6, IL-10, IL-13, and TGFβ [18,21] were synthesized by Integrated DNA Technologies (Coralville, IA, USA), and the primer sequences along with the gene-specific annealing temperature are given in Table 1. Using the formula to quantify the relative amount of gene expression that is *E* of the target standard curve ^(Cp of target calibrator) − (Cp of target samples)^ / *E* of reference standard curve ^(Cp of reference samples) − (Cp of reference calibrator)^, the expression levels of target genes were calculated with the housekeeping gene, β-actin reference gene, as described previously [22,23].

### 2.6. Statistical Analysis

All the data were analyzed using GraphPad Prism V9.4 (GraphPad software, San Diego, CA, USA). For gross lesion scores analysis, a Kruskal–Wallis test, followed by Dunn’s multiple comparison test was used since the data were not normally distributed. Statistical comparisons were made between the experimental groups (control, CP5, CP18, and CP26). For gene expression and body weight gain data, normally distributed data were analyzed by the parametric tests, one/two-way ANOVA followed by Turkey’s multiple comparisons tests, while the nonparametric test, Kruskal–Wallis test, was used when the data were not normally distributed. Data were presented as mean ± standard error of the mean (SEM) and the level of statistical significance considered was at *p* < 0.05.

## 3. Results

Three *C. perfringens* strains, CP5 (*netB^+^ tpeL^−^*), CP18 (*netB^+^ tpeL^+^*), and CP26 (*netB^+^ tpeL^+^*), were tested for their ability to induce NE and their effect on immune system gene expression in mucosal and systemic lymphoid organs in broiler chickens. The results below show the intracloacally administered food dye distribution in the intestine, followed by a description of results related to the gross intestinal lesions, body weight gains, and changes in the gene expression of IFNγ, IL-1β, IL-4, IL-6, IL-10, IL-13, TLR-21, and TGFβ cytokines in *C. perfringens*-infected and uninfected chickens.

### 3.1. Intracloacal Inoculation with the Food Dye

**Figure 1:**
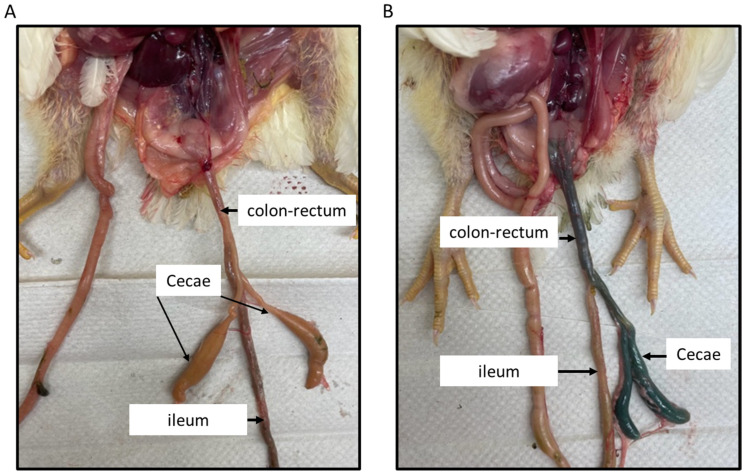
**Intracloacal inoculation with the food dye.** About 1 mL of the food dye (black) was intracloacally administered to 3-week-old broiler chickens, along with the PBS control. Birds were necropsied 1hr post-inoculation and the large and small intestinal segments were observed for the presence of black dye. The picture (**A**) shows different parts of the intestine in a birds inoculated with PBS, while the black dye deposition in the regions of rectum–colon and ceca is shown in picture (**B**). No dye was observed in the ileum.

Birds necropsied at 1hr post-inoculation were examined for the distribution of food dye in the small and large intestinal segments of 3-week-old broilers inoculated intracloacally. Figure 1 shows different parts of the intestine (colon–rectum, cecum, and ileum) in a bird inoculated with PBS (Figure 1A), while the black food dye deposition in the regions of rectum–colon and ceca, with no dye observed in the ileum, is depicted in Figure 1B.

### 3.2. Gross Pathology and Body Weight Gains

**Figure 2:**
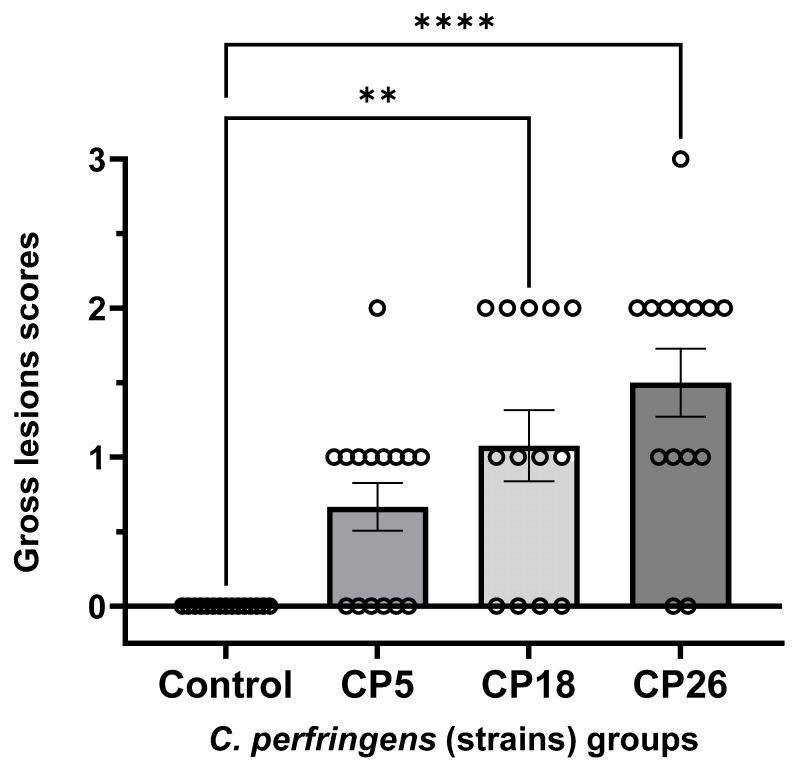
**Gross pathology lesion scores in the small intestines of chickens intracloacally infected with *C. perfringens* strains**. One-day-old commercial broiler chickens were fed an antibiotic-free diet and challenged with one of three different *C. perfringens* strains along with the uninfected control via intracloacal inoculation on day 18 of age, administered twice daily for 4 consecutive days, followed by necropsy on the 22nd day of age. The gross lesions in the small intestine (duodenum to ileum) were scored. Individual animal scores corresponding to each of the groups along with the mean lesion scores are shown. Statistical significance between the treatment groups is indicated above the standard error of mean bars. Control—uninfected; CP—*C. perfringens* strain. Asterisks above the standard error of mean bars between groups indicate the differences were statistically significant at *p* < 0.01 (**) or *p* < 0.0001 (****).

As shown in Figure 2, scoring of gross lesions in the small intestine of chickens challenged with three different strains of *C. perfringens* showed that birds receiving CP18 and CP26 inoculations had significantly higher (*p* < 0.05) lesion scores compared to the uninfected control groups, suggesting that these strains were virulent. Although not statistically significant, the CP26-infected group had relatively more birds with scores of 2 or 3 when compared to those in the CP18-infected group. Specifically, the CP26-infected group had seven birds with a score of 2 and one bird with a score of 3, while the CP18-infected group had five birds with a score of 2 and none of the birds had a score of 3. The uninfected control birds did not develop lesions, while 14 out of 15 birds infected with CP5 had lesions scores of either 0 or 1; however, no significant (*p* < 0.05) differences were observed between the control and CP5-infected groups, suggesting CP5 was an avirulent strain. No significant (*p* < 0.05) differences were observed between the CP5 and CP18/CP26 groups.

**Figure 3:**
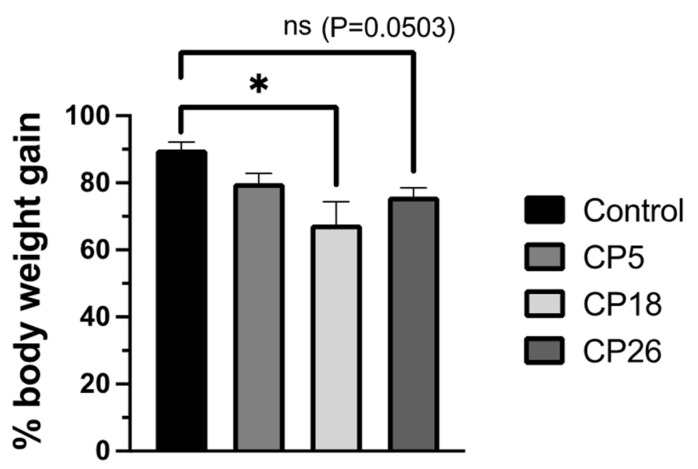
**Effect of *C. perfringens* on body weight gains.** One-day-old commercial broiler chickens were fed an antibiotic-free diet and challenged with one of three different *C. perfringens* strains, along with the uninfected control, via intracloacal inoculation on day 18 of age, administered twice daily for 4 consecutive days, followed by necropsy on the 5th day. The body weights were collected on day 17 (pre-challenge) and 22 (post-challenge, prior to necropsy) and the percent body weight gain was calculated. Statistical significance between the treatment groups is indicated above the standard error of mean bars. Control—uninfected; CP—*C. perfringens* strain. An asterisk above the standard error of mean bars between groups indicate the differences were statistically significant at *p* < 0.05; ns- non-significant.

The changes in the % body weight gain were determined during the course of the infection challenge; CP18 inoculation resulted in a decrease (*p* < 0.05) in body weight gain compared to the uninfected control birds (Figure 3). The weight gain in the CP26-infected group compared to the control was also reduced (*p* = 0.0503). No significant changes were observed between the CP5 group and the control group.

### 3.3. Immune System Gene Expression in Lymphoid Tissues

#### 3.3.1. Cecal Tonsils

**Figure 4:**
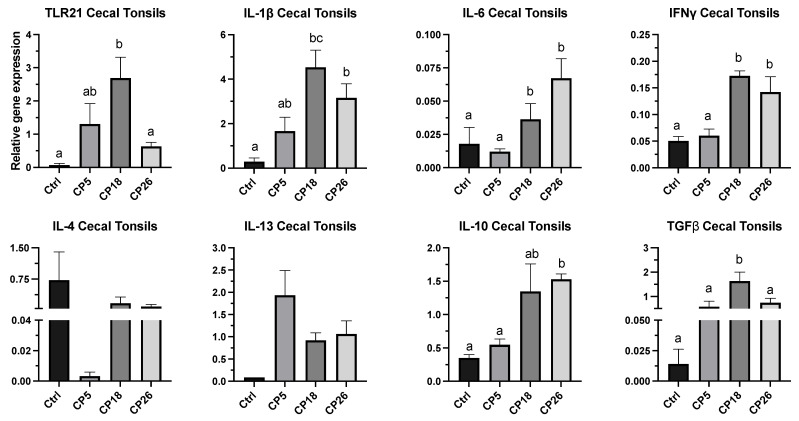
**Effect of *C. perfringens* on immune system gene expression in the cecal tonsils of broiler chickens.** One-day-old commercial broiler chickens were fed an antibiotic-free diet and challenged with one of three different *C. perfringens* strains along with the uninfected control via intracloacal inoculation on day 18 of age, administered twice daily for 4 consecutive days, followed by necropsy on day 22. Cecal tonsils were collected in RNAlater for RNA extraction and cDNA synthesis. Real-time PCR to quantify the expression of IL-1β, IL-4, IL-6, IL-10, IL-13, IFNγ, TLR-21, and TGFβ genes was performed, along with the housekeeping gene (β-actin). The expression levels are shown as relative to β-actin. Different letters above the standard error of mean bars indicate significant differences (*p* < 0.05) between the groups. Ctrl—Control (uninfected); CP—*C. perfringens* strain.

As shown in Figure 4, the expression of IL-1β, IL-6, and IFNγ genes in CP18- and CP26-infected birds was higher (*p* < 0.05) than that in the uninfected control. Additionally, the expression of IL-6 and IFNγ genes in both CP18- and CP26-infected birds was higher (*p* < 0.05) than that in the CP5 group. The expression of TLR-21 and TGFβ was higher (*p* < 0.05) in the CP18-infected birds compared to the control and CP26 groups, whereas the TGFβ transcription in the CP18-infected birds was also higher (*p* < 0.05) than the CP5 group. Furthermore, CP26 infection induced a higher (*p* < 0.05) expression of IL-10 compared to the control and CP5 groups. No significant changes were observed in the transcription of IL-4 and IL-13 between any of the treatment groups.

#### 3.3.2. Bursa of Fabricius

**Figure 5:**
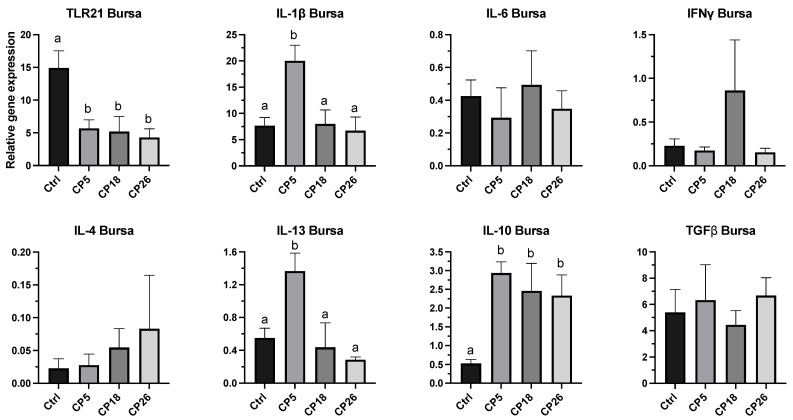
**Effect of *C. perfringens* on immune gene expression in the bursa of Fabricius of broiler chickens.** One-day-old commercial broiler chickens were fed an antibiotic-free diet and challenged with one of three different *C. perfringens* strains along with the uninfected control via intracloacal inoculation on day 18 of age, administered twice daily for 4 consecutive days, followed by necropsy on day 22. Bursa were collected in RNAlater for RNA extraction and cDNA synthesis. Real-time PCR to quantify the expression of IL-1β, IL-4, IL-6, IL-10, IL-13, IFNγ, TLR-21, and TGFβ genes was performed along with the housekeeping gene (β-actin). The expression levels are shown as relative to β-actin. Different letters above the standard error of mean bars indicate significant difference (*p* < 0.05) between the groups. Ctrl—Control (uninfected); CP—*C. perfringens* strain.

As shown in Figure 5, the expression of IL-1β and IL-13 genes in CP5-infected birds was higher (*p* < 0.05) compared to the control, CP18, and CP26 groups. Additionally, the expression of IL-10 in all infected groups was increased (*p* < 0.05), while the TLR21 expression was decreased (*p* < 0.05) in all the infected groups when compared to the uninfected control. No significant changes were observed in the transcription of IL-6, IFNγ, IL-4, and TGFβ between any of the treatment groups.

#### 3.3.3. Harderian Gland

**Figure 6:**
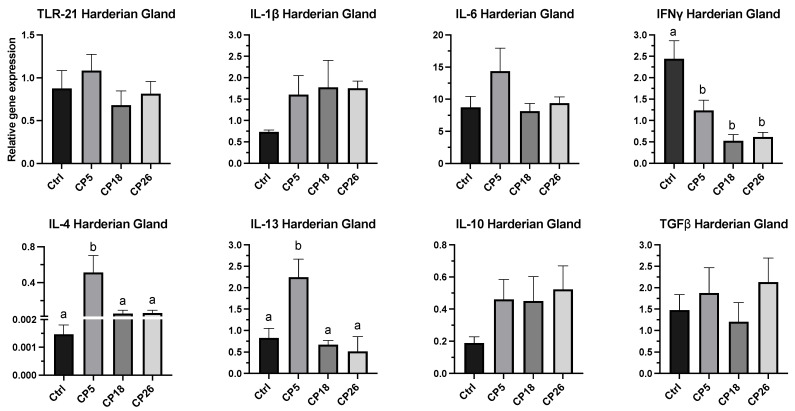
**Effect of *C. perfringens* on immune system gene expression in the Harderian gland of broiler chickens.** One-day-old commercial broiler chickens were fed an antibiotic-free diet and challenged with one of three different *C. perfringens* strains along with the uninfected control via intracloacal inoculation on day 18 of age, administered twice daily for 4 consecutive days, followed by necropsy on day 22. Harderian glands were collected in RNAlater for RNA extraction and cDNA synthesis. Real-time PCR to quantify the expression of IL-1β, IL-4, IL-6, IL-10, IL-13, IFNγ, TLR-21, and TGFβ genes was performed along with the housekeeping gene (β-actin). The expression levels are shown as relative to β-actin. Different letters above the standard error of mean bars indicate significant difference (*p* < 0.05) between the groups. Ctrl—Control (uninfected); CP—*C. perfringens* strain.

As shown in Figure 6, the expression of IL-4 and IL-13 in CP5-infected birds was increased (*p* < 0.05) compared to the control, CP18, and CP26 groups. The expression of IFNγ in all infected groups was decreased (*p* < 0.05) compared to the control. No significant changes were observed in the expression of TLR-21, IL-1β, IL-6, IL-10, or TGFβ between the infected and control groups.

#### 3.3.4. Spleen

**Figure 7:**
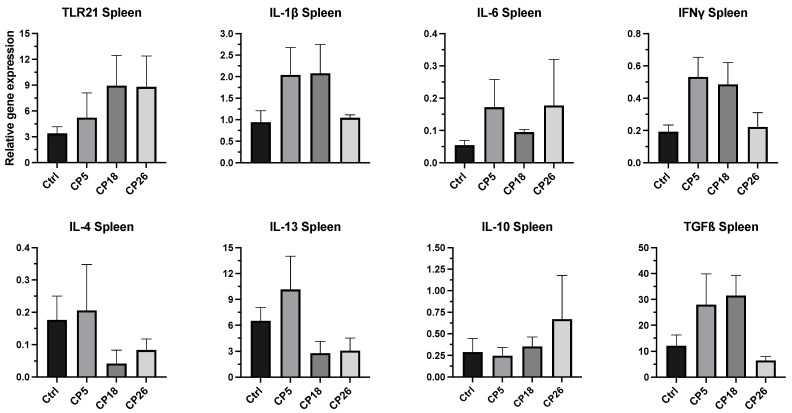
**Effect of *C. perfringens* on immune gene expression in the spleen of broiler chickens.** One-day-old commercial broiler chickens were fed an antibiotic-free diet and challenged with one of three different *C. perfringens* strains along with the uninfected control via intracloacal inoculation on day 18 of age, administered twice daily for 4 consecutive days, followed by necropsy on day 22. Spleens were collected in RNAlater for RNA extraction and cDNA synthesis. Real-time PCR to quantify the expression of IL-1β, IL-4, IL-6, IL-10, IL-13, IFNγ, TLR-21, and TGFβ genes was performed along with the housekeeping gene (β-actin). The expression levels are shown as relative to β-actin. Different letters above the standard error of mean bars indicate significant differences (*p* < 0.05) between the groups. Ctrl—Control (uninfected); CP—*C. perfringens* strain.

As depicted in Figure 7, no significant changes in the expression of any of the cytokine or TLR genes were observed between the infected and control groups.

In addition to the figures presented here, a summary highlighting the statistically significant (*p* < 0.05) changes in the expression of immune genes in chickens infected with *C. perfringens* strains when compared to uninfected control is given in Table 2.

## 4. Discussion

Both clinical and subclinical forms of NE are negatively impacting the poultry production economy in the current era of ‘no antibiotic ever’ farming. Understanding *C. perfringens*–host interactions will help devise novel prophylactic and therapeutic strategies for disease control. For studying NE pathogenesis and immunity, the experimental infection models have routinely used oral gavage as the route for inoculating chickens with *C. perfringens* [7,12]. In case of certain bacterial infections such as those caused by *Salmonella* spp. [24] or *Campylobacter jejuni* [25] in poultry, the cloacal route has also been suggested as a potential route of entry for these pathogens. A previous study showed that a cloacal application of fluorescein-labeled polystyrene beads can facilitate the beads to reach the intestinal segments, namely the colon–rectum, cecum, and ileum, possibly through the process of ‘cloacal drinking’ [26]. Exploring the intracloacal route as another possible route of *C. perfringens* infection will further our understanding of NE induction and help delineate the immune mechanisms involved in limiting disease development and severity, if any. In an experimental oral infection model of clinical NE in broiler chickens, we have recently shown that the immune responses during NE are spatially regulated in lymphoid tissues such that the inflammatory responses against *C. perfringens* depended on the virulence of the strain [4]. In the present study, we sought to use three strains of *C. perfringens*, which were virulence-characterized in the previous study [4] as avirulent CP5 (*netB^+^ tpeL^−^*), and virulent CP18 (*netB^+^ tpeL^+^*) and CP26 (*netB^+^ tpeL^+^*), to investigate the outcome of their intracloacal inoculation on the nature of NE severity and its associated immune responses in lymphoid tissues. The findings revealed two important observations: (1) Intracloacal infection of birds with virulent strains produced milder or less severe intestinal lesions compared to our earlier observations in the orally infected chickens and this observation was coupled with reduced body weight gain, suggesting a subclinical-grade NE. (2) While an inflammatory response against virulent strains was observed in the CT of infected birds, a marked anti-inflammatory or immunomodulatory response against *C. perfringens* was evident in CT, bursa, and HG, suggesting a spatial regulation of inflammation in these mucosal lymphoid tissues during subclinical NE.

The pathogenesis of NE involves many factors, including intestinal predisposition by dietary changes or coccidia infections, and importantly, the virulence nature of *C. perfringens* strains. While clinical NE is characterized by severe intestinal damage and mortality, the subclinical form is usually associated with poor body weight gain and milder intestinal lesions. The disease is manifested when certain *netB^+^* virulent strains proliferate to high numbers and produce toxins, resulting in ulcerative and necrotic lesions in the small intestine [7]. The virulence factors include various tissue or mucus degrading enzymes and adhesins, and, importantly, the toxins, of which NetB, alpha-toxin, and TpeL all contribute to virulence [27,28]. In a previous study, we used *C. perfringens* strains (CP18 and CP26) to show that the carriage of the *tpeL* toxin gene in some *netB^+^* strains (CP26) can enhance *C. perfringens* virulence when inoculated orally [4], which was also in agreement with others [2,3]. Interestingly, in the present study, inoculation with the same strains (CP18 and CP26) intracloacally resulted in markedly less severe intestinal lesions. A case in point was that the CP26 strain, which produced extensive necrotic lesions when administered orally [4], could only produce mild ulcerative lesions when administered intracloacally in the present study. It was evident that the intracloacal route which allowed the inoculum to reach the ceca and not beyond (Figure 1) had an impact on the outcome of virulent *C. perfringens*-induced NE severity for the following two possible reasons: (1) The ceca being enriched with diverse microbiota populations, including *C. butyricum*, generally render pathogens devoid of a local niche to colonize, overgrow, and produce disease through various mechanisms, including competitive exclusion, increased mucus production, and downregulation of inflammation [29,30]. (2) Although the virulent *C. perfringens* secrete NetB and TpeL-like cytolytic and pore-forming toxins, it is possible that their toxin production may either be diminished by the local cecal–colorectal environment [31,32] or that they were devoid of cellular targets for cytolysis. On the contrary, *C. perfringens* entry via the oral route likely encounters a relatively lower microbial competition to colonize, and enhanced access to nutrients for their growth and toxin production, thus facilitating a conducive environment for the pathogen adaptation [7,28]. Taken together, our findings that showed an intracloacal inoculation of chickens with virulent *C. perfringens* can result in milder NE lesions and a reduction in body weight gain suggest that this model can be used to induce a subclinical infection, which may be suitable for future studies aimed at evaluating the efficacy of research-based novel feed additives, direct-fed microbials, or vaccines.

Intestinal inflammation is often considered the key to a successful enteric disease establishment. While some pathogens avoid host recognition through immunoevasive mechanisms, some use induction of inflammatory responses as a means to cause excessive self-inflicted host tissue damage, thereby creating an opportunity to survive and proliferate [33]. For example, certain human strains of *Shigella dysenteriae* [34], *Staphylococcus aureus,* or *Streptococcus pyogenes* [35] are shown to induce robust inflammatory responses and such a response was linked to those strains that carried accessory virulence genes in their genomes. Along similar lines, our previous study showed that virulent *C. perfringens* strains (CP18 and CP26), when given orally to broiler chickens, showed an increase in the expression of IL-1β, IL-6, and IFN-γ pro-inflammatory cytokine genes in the local lymphoid tissue (CT), and furthermore, the CP26 strain was also found to induce a splenic inflammatory response, suggesting the likely systemic effect of toxins and other virulence molecules secreted by the very virulent strains during severe NE infections [4]. Studies by other groups have also demonstrated elevated transcriptional levels of pro-inflammatory cytokines in the intestinal tissues using similar oral infection models, as reviewed previously [6,9]. For example, broiler chickens coinfected with *E. maxima* and virulent *C. perfringens* induced an increased intestinal gene expression of pro-inflammatory cytokines (IL-1β, IFNγ, IL-17, IL-8, and others) [16,36], while in vitro stimulation of chicken intestinal epithelial cells with virulent *C. perfringens* or purified alpha-toxin led to an increased transcription of IL-6, IL-8, inducible nitric oxide synthase (iNOS), tumor necrosis factor (TNF), and nuclear factor kappa-B (NFkB) genes [37]. In support of these findings, macrophages have been shown as the likely source of such mucosal inflammatory responses [18,38] [Kulkarni, et. al., 2023; unpublished]. To this end, the present study found that the broiler birds infected intracloacally with virulent CP18 and CP26 strains showed a significantly increased CT expression of IL-1β, IL-6, and IFNγ compared to the uninfected control and avirulent CP5-infected groups. This observation was in support of our previous studies that host inflammatory responses are often mounted against the virulent, but not avirulent, strains [4,18]. However, this response in CT appeared to be highly regulated when birds were infected intracloacally, as demonstrated by an increased expression of anti-inflammatory/ immunoregulatory cytokines or downregulation of expression of pro-inflammatory cytokines in the mucosal lymphoid tissues, including CT, bursa, and HG. For example, the expression of IL-10 and TGFβ genes was found to be significantly increased in the CT and/or bursa of birds infected with virulent *C. perfringens* compared to the uninfected and CP5-infected birds. The expression of anti-inflammatory cytokine genes in both CT and bursa may explain, at least in part, the concurrent downregulation of the IFNγ expression in the HG. Importantly, no significant changes in the splenic expression of cytokine genes between infected and control birds were observed. Although not investigated in this study, these observations reinforce our speculation that intracloacally-administered *C. perfringens* induces local anti-inflammatory responses that counteract the inflammation associated with NE, consequently leading to a reduction in the proliferation of *C. perfringens* and toxin production to a level that may not be sufficient to induce systemic responses [4]. Another likely reason for this effect is the presence of a diverse population of microbiota in the distal segments of the intestine that help maintain a steady-state immune homeostasis under healthy conditions in birds [39]. This is because the mucosal microbiota, the majority of which are colonized in the cecum, are known to regulate inflammatory responses locally as well as distantly via upregulating the production of IL-10 and/or TGFβ in the mucosal tissues and its associated lymphoid organs (CT and bursa) operating through a common mucosal system axis [40,41,42]. The immunomodulatory effects of microbiota are mediated by immune system cells, such as CD4+ regulatory T cells and CD4+ T helper (Th)-2 cells that secrete IL-10, TGFβ, IL-4, and IL-13 cytokines, which are known for their role in the regulation of inflammation, as well as the promotion of B cell proliferation and antibody secretion [41,43]. To this end, the present study also found a transcriptional upregulation of IL-4 and/or IL-13 in the bursa and HG of *C. perfringens*-infected chickens. It is also noteworthy that the HG, an important lymphoid tissue located supraorbital in chickens, is a part of the common mucosal system [44] and a recent study in chickens showed that HG can downregulate inflammatory responses via expression of microRNAs, such as mir-1764 [45].

Another intriguing observation in the present study was that the birds infected with the virulent CP18 strain had a significantly elevated CT transcription of TLR21, a bacterial DNA sensing receptor in the avian immune system cells, while the expression of this receptor in the bursa of chickens infected with all three strains was significantly downregulated when compared to the uninfected control. An increased TLR21 transcription in the CT suggests a possible involvement of this receptor in the recognition of virulent *C. perfringens* by the immune cells, which is in agreement with our previous reports [4,18]. However, the downregulated expression of the TLR21 gene in the bursa of *C. perfringens*-infected chickens possibly suggests a negative regulatory mechanism of TLR signaling in the immune cells residing within or trafficking through the bursa to regulate intestinal inflammation [46]. In partial support of this notion, a previous report investigating *Salmonella* infection in chickens found a regulated expression of TLR4 in eosinophils and mast cells that were recruited to bursa post-infection [47]. Nevertheless, further investigations are needed to ascertain the specific role as well as the mechanism of TLR21 expression in mediating the induction and regulation of innate immune responses during *C. perfringens*-induced NE in chickens.

## 5. Conclusions

In summary, the findings of the present study showed that intracloacal inoculation of broiler chickens with virulent strains of *C. perfringens* can produce a subclinical grade NE infection, characterized by mild ulcerative intestinal lesions and reduced body weight gain. Furthermore, intracloacal *C. perfringens* inoculation seems to induce a highly regulated inflammatory response in mucosal lymphoid organs. This study offers a new subclinical NE model that can be used in future research to decipher the mechanisms of immune activation and regulation during infection with *C. perfringens* and to assess the efficacy of vaccines and therapeutics against a subclinical form of NE.

## Figures and Tables

**Table 1:** Primer sequences used in real-time PCR assay.

Gene	Primer Sequence (F-Forward; R-Reverse)	Annealing Temperature (°C)	GenBank Accession Number
IFNγ	F: 5′-ACACTGACAAGTCAAAGCCGCACA-3′ R: 5′-AGTCGTTCATCGGGAGCTTGGC-3′	60	X99774
IL-4	F: 5′-TGTGCCCACGCTGTGCTTACA-3′ R: 5′-CTTGTGGCAGTGCTGGCTCTCC-3′	60	AJ621249.1
IL-6	F: 5′-CGTGTGCGAGAACAGCATGGAGA-3′ R: 5′-TCAGGCATTTCTCCTCGTCGAAGC-3′	60	NM_204628.1
IL-10	F: 5′-AGCAGATCAAGGAGACGTTC-3′ R: 5′-ATCAGCAGGTACTCCTCGAT-3′	55	AJ621614
IL-1β	F: 5′-GTGAGGCTCAACATTGCGCTGTA-3′ R: 5′-TGTCCAGGCGGTAGAAGATGAAG-3′	64	AJ009800
IL-13	F: 5′-ATCCTGCTGGAGCCCATTCAGAG-3′ R: 5′-TTGCTCTTCATCAGGAGGCCACT-3′	60	NM_204278.1
TLR-21	F: 5′-CCTGCGCAAGTGTCCGCTCA-3′ R: 5′-GCCCCAGGTCCAGGAAGCAG-3′	60	NM_001030558.1
TGFβ	F: 5′-CGGCCGACGATGAGTGGCTC-3′ R: 5′-CGGGGCCCATCTCACAGGGA-3′	60	M31160.1
β-actin	F: 5′-CAACACAGTGCTGTCTGGTGGTA-3′ R: 5′-ATCGTACTCCTGCTTGCTGATCC-3′	58	X00182

**Table 2:** Summary of changes in the immune system gene expression in the lymphoid tissues of chickens infected with *C. perfringens* strains compared to uninfected control group.

	Lymphoid Tissues *								
	Cecal Tonsil			Bursa			Harderian Gland		
	CP5	CP18	CP26	CP5	CP18	CP26	CP5	CP18	CP26
TLR21	=	+	=	-	-	-	=	=	=
IL-1 β	=	+	+	+	=	=	=	=	=
IL-6	=	+	+	=	=	=	=	=	=
IFNγ	=	+	+	=	=	=	-	-	-
IL-4	=	=	=	=	=	=	+	=	=
IL-13	=	=	=	+	=	=	+	=	=
IL-10	=	=	+	+	+	+	=	=	=
TGFβ	=	+	=	=	=	=	=	=	=

* Data for the spleen are not shown here since no significant changes in the immune gene expression were observed between the infected and control groups. A ‘+’ sign denotes increased (*p* < 0.05) and a ‘-’ for decreased (*p* < 0.05) gene expression level compared to uninfected control, while a ‘=’ denotes no significant changes compared to uninfected control.

## Data Availability

Research data are available upon request from R.R.K.
